# The Barley Glycosyltransferase Gene *KOB1* Implicated in β-Glucan Biosynthesis by a Genome-Wide Association Study

**DOI:** 10.3390/plants14213269

**Published:** 2025-10-26

**Authors:** Guangyou Wan, Zonghui Lu, Ruibin Ren, Dan Zhang, Erjing Si, Lixia Yao, Juncheng Wang, Huajun Wang, Xiaole Ma, Hong Zhang, Lirong Yao, Baochun Li, Qijun Bao, Yaxiong Meng

**Affiliations:** 1 Department of Crop Genetics and Breeding, College of Agronomy, Gansu Agricultural University, Lanzhou 730070, China; guangyouwan@163.com (G.W.); zonghuilu23@163.com (Z.L.); ruibinren@163.com (R.R.); dangzhang0811@163.com (D.Z.); sierjing@163.com (E.S.); lixiayao23@163.com (L.Y.); wangjc@gsau.edu.cn (J.W.); wanghj@gsau.edu.cn (H.W.); maxl@gsau.edu.cn (X.M.); 18851095887@163.com (H.Z.); ylr0384@163.com (L.Y.); 2 State Key Lab of Aridland Crop Science, Gansu Key Lab of Crop Improvement and Germplasm Enhancement, Lanzhou 730070, China; libc@gsau.edu.cn; 3 Department of Botany, College of Life Sciences and Technology, Gansu Agricultural University, Lanzhou 730070, China; 4 Institute of Economic Crops and Beer Raw Materials, Gansu Academy of Agricultural Sciences, Lanzhou 730070, China

**Keywords:** barley, (1,3;1,4)-beta-glucan, candidate gene, functional verification

## Abstract

β-glucan, a crucial trait in barley breeding programs, serves as a quality determinant of products intended for both human consumption and animal feed. Although genes involved in β-glucan synthesis have been reported, the genetic mechanisms regulating its accumulation in barley grain remain underexplored. In this study, we functionally characterized *KOB1*, a candidate gene identified from a genome-wide association study (GWAS) on barley seed β-glucan content, which encodes a glycosyltransferase. Haplotype analysis showed that haplotype E was associated with significantly elevated grain β-glucan levels compared to other haplotypes. Furthermore, overexpression of *KOB1* in rice led to a significant increase in grain β-glucan content relative to the wild-type Zhonghua 11, confirming its critical role in β-glucan biosynthesis. Our findings establish the glycosyltransferase gene *KOB1* as a valuable genetic resource for molecular breeding programs aimed at improving grain β-glucan content.

## 1. Introduction

As a globally cultivated staple crop, barley (*Hordeum vulgare* L.) ranks as the world’s fourth-largest cereal and is significantly important for both human consumption and animal feed [1,2]. This multifunctional grain has gained scientific attention particularly for its nutritional profile, with (1,3;1,4)-beta-glucan (β-glucan) emerging as a key bioactive component that substantially influences grain quality [3]. In comparison with other cereal crops, including wheat, maize, and rice, barley is distinguished by its high β-glucan content, which generally range from 2% to 9% [4,5]. β-Glucans are polysaccharides present in the cell walls of barley, with the highest levels of β-glucans found within the endosperm and aleurone layer of the seeds [6,7]. When barley is used as food for humans, β-glucan improves immune system function, lowers the glycemic index and cholesterol levels in the blood, and helps reduce the risk of obesity and cardiovascular disease [8]. Therefore, increasing the β-glucan content in the grain is one of the main goals of barley breeding programs. Given the promising applications, the barley seed β-glucan in functional food development and health product consumption, more attention is being paid to the molecular regulatory mechanisms of barley β-glucan synthesis and accumulation [9].

The role of cellulose synthase-like CSLF gene family members in β-glucan biosynthesis was confirmed through heterologous expression of the respective rice genes [10]. Several subsequent studies have shown that two gene families, *CslF* and *CslH*, regulate β-glucan synthesis [11]. Some researchers have also found that changes in starch synthesis genes lead to changes in β-glucan content [12]. Genome-wide association study (GWAS) has become an important tool for studying genetic loci associated with critical agronomic traits in crops [13]. Pioneering research utilizing GWAS approaches identified significant single nucleotide polymorphisms (SNPs) associated with grain β-glucan content [14,15,16,17]. Through multi-environment GWAS analysis of 119 barley accessions, the study identified 10 candidate genes that potentially regulate β-glucan biosynthesis, compartmentalization, and catabolism during grain development [18]. Combined, GWAS analysis of β-glucan content across diverse barley cultivars and functional validation of candidate genes identified seven members of the *Csl* gene family as involved in β-glucan synthesis [19,20]. Members of the *CslF* gene family play a crucial role in determining β-glucan content, and overexpression of the *CslF6* gene, controlled by an endosperm-specific promoter, significantly increases β-glucan content [21]. Understanding linkage disequilibrium (LD) is fundamental to genetic analyses such as genome-wide association studies and marker-assisted breeding, as it is powerful for revealing patterns of genetic variation and identifying markers linked to traits of interest [22]. Researchers identify candidate genes by analyzing LD blocks surrounding significant SNPs or by applying genome-wide LD approaches [23]. Du et al. identified favorable haplotypes related to flag leaf shape through LD block analysis of candidate genes related to flag leaf shape in rice [24]. With the rapid development of molecular biology, many molecular biology approaches have emerged to verify gene functions and explore their mechanisms of action. Yuan et al. demonstrated in a transgenic assay that a candidate gene (*OsFAH2*) controlled seed storage tolerance and antioxidant capacity, and that its overexpression reduced lipid peroxidation and increased seed storage tolerance [25]. Although quantitative trait loci (QTLs) and candidate genes for β-glucan content have been identified through linkage mapping and GWAS, a complete understanding of their biological functions often requires direct functional evidence [12]. Our study provides a foundational resource and prioritized gene list to bridge this gap, enabling future research that integrates genomic and gene-editing approaches to fully elucidate the regulatory mechanisms of β-glucan synthesis.

In this study, we performed a GWAS on 238 barley accessions to identify candidate genes for grain β-glucan content and to provide insights into its molecular regulation. Our analysis highlighted genes involved in β-glucan metabolism, including putative regulators of grain development. Functional evidence from transgenic overexpression of the glycosyltransferase-encoding gene *KOB1* showed that it significantly increases β-glucan content.

## 2. Results

### 2.1. Genome-Wide Association Analysis and Candidate Gene Prediction

**Figure 1:**
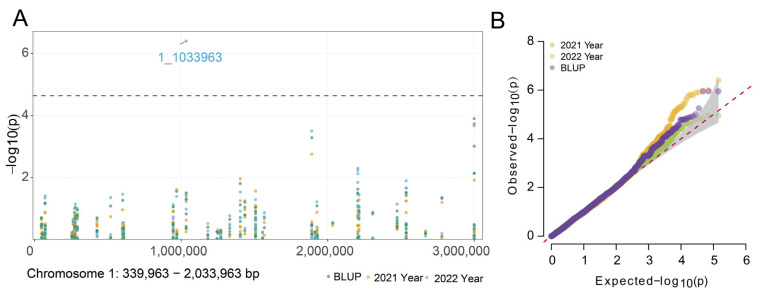
Genome-wide association study of β-glucan content in barley seeds. (**A**) A local Manhattan plot on chromosome 1H shows the distribution of trait-marker associations. The dashed red line indicates the genome-wide significance threshold (−log_10(P)_ = 4.64), determined by the Bonferroni correction. Peaks surpassing this threshold represent potential quantitative trait loci (QTLs), with the most significant SNP marker labeled. (**B**) QQ plot based on PCA model, the different colored points in the graph represent different years and BLUP values.

We performed a genome-wide association study (GWAS) using 114,652 high-quality SNP markers that were retained after quality control processing. The significance threshold for SNP associations was established using the (1/Ne) criterion. Evaluation of QQ plots indicated that the principal components analysis (PCA) model was superior for controlling false positives and was therefore selected for the GWAS analysis (Figure 1B). Using β-glucan content data along with corresponding best linear unbiased prediction (BLUP) values derived from barley grains over two consecutive growing seasons (2021 and 2022), we identified several significant SNP loci associated with this trait. The most significant association was observed for SNP 1_1033963 on chromosome 1H, which reached a −log_10(P)_ value of 4.64 (Figure 1A). Annotation of the genomic regions spanning 300 kb around each significant marker identified 37 candidate genes, among which were several transcription factors and enzymes potentially implicated in the biosynthesis of β-glucan in barley grains (Table S2).

### 2.2. Identification and Analysis of Candidate Genes

**Figure 2:**
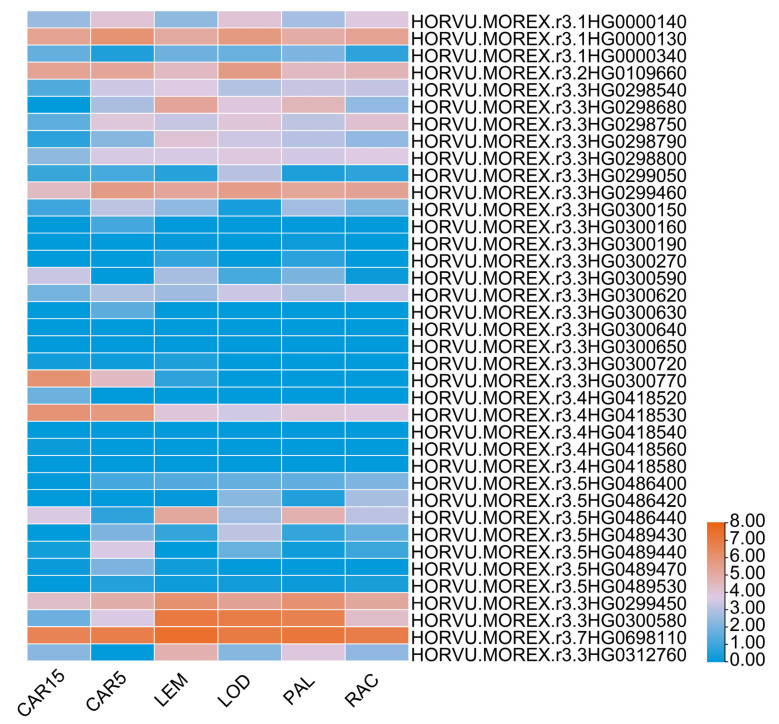
Heatmap of transcript expression for thirty-seven candidate genes and two known β-glucan biosynthesis-related genes (*HvCslF6*, *HvBGlu3*). For each period, the transcript expression values were log-transformed, ranging from 0 to 8. Red indicates high expression, and blue indicates low expression. CAR15: bracts removed (15 DPA); CAR5: bracts removed (5 DPA); LEM: lemma (6 weeks PA); LOD: lodicule (6 weeks PA); PAL: palea (6 weeks PA); RAC: rachis (5 weeks PA).

Genes exhibiting high and sustained expression during the critical period of β-glucan accumulation [26,27,28] were selected from the initial set of 37 candidates, based on transcriptome data across seed developmental stages. The final three candidate genes were identified through integration of functional annotation and supporting evidence from relevant published studies. Among them, the gene *HORVU.MOREX.r3.1HG0000140*, located on chromosome 1H, encodes barley glycosyltransferase. The genes *HORVU.MOREX.r3.3HG0298680* and *HORVU.MOREX.r3.3HG0300770* encode the MYB transcription factor and ABI5 transcription factor, respectively (Table S2). Notably, the candidate gene *HORVU.MOREX.r3.1HG0000140* exhibited high expression levels during critical stages of barley grain development, including the CAR5 stage (bracts removed at 5 days post-anthesis, DPA), LOD stage (lodicule at 6 weeks post-anthesis), and RAC stage (rachis at 5 weeks PA). Moreover, it displayed a similar expression profile to the key β-glucan synthase gene *HvCslF6* (Figure 2). The remaining genes (*HORVU.MOREX.r3.1HG0000130*, *HORVU.MOREX.r3.2HG0109660*, *HORVU.MOREX.r3.4HG0418530*) exhibit expression patterns comparable to *HvCslF6* (5–15 DPA), although their functional annotations and reported studies suggest no established link to glucan biosynthesis. We also found that the expression pattern of *HORVU.MOREX.r3.1HG0000140* was divergent from that of the confirmed negative regulator of β-glucan content, *HvBGlu3*. The gene *HvBGlu3* showed elevated expression during the LEM (lemma, 6 weeks post-anthesis) and PAL (palea, 6 weeks post-anthesis) stages (Figure 2). Gene annotation and literature analysis indicated that *HORVU.MOREX.R3.1HG0000140* is homologous to *AT3G08550* in Arabidopsis thaliana, which encodes a glycosyltransferase involved in cell elongation and cellulose biosynthesis. Consistent with this homology, *HORVU.MOREX.R3.1HG0000140* is predicted to encode a glycosyltransferase of the GT family. Its protein architecture features a conserved glycosyltransferase domain, characteristic of processive enzymes that catalyze polysaccharide chain elongation. In Arabidopsis, the *KOB1* protein localizes to the plasma membrane, where it is essential for normal cellulose synthesis during cell elongation [29]. Protein–protein interaction prediction analysis using the STRING database (https://cn.string-db.org/, accessed on 15 July 2025) further suggests that *AT3G08550* may interact with several regulators of seed development. Given the functional homology of glycosyltransferases across plant species, we hypothesized that its barley ortholog might similarly influence β-glucan biosynthesis.

Finally, the gene *HORVU.MOREX.R3.1HG0000140* encoding glycosyltransferase near the most significant site 1_1033963 was selected for functional studies based on gene annotation, all available studies and expression analysis. On the basis of functional annotation and consistent evidence from previous studies, we therefore assign the name *KOB1* to the candidate gene *HORVU.MOREX.R3.1HG0000140*.

### 2.3. Candidate Gene KOB1 with Significant SNP 1_1033963 Locus

**Figure 3:**
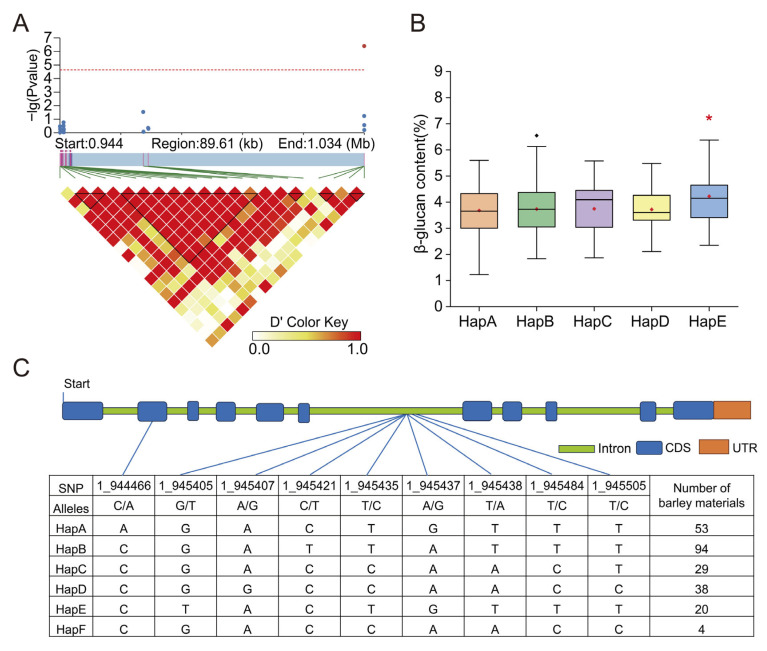
Candidate gene analysis. (**A**) Regional association plot and linkage disequilibrium (LD) heatmap across the *KOB1* locus. The left y-axis and data points represent the genome-wide association strength −log(P) for each SNP, with the blue and red dots denoting SNPs in the linkage disequilibrium region and the significant SNP (1_1033963), respectively. The red dashed line indicates the significance threshold. The underlying color matrix depicts the pairwise LD, measured by D′, between SNPs. D′ values range from 0 (white, no LD) to 1 (red, complete LD). The black box represents a region of high linkage disequilibrium. (**B**) Haplotype analysis of β-glucan content of the candidate gene *KOB1*. Haplotypes with fewer than 5 varieties are not shown. The black points in the graph represent outliers, the red square points represent the mean, and the asterisks represent significance (* *p* < 0.05) (**C**) Genetic variation and gene structure of *KOB1*. Six major haplotypes of *KOB1* identified based on nine SNPs across all assessed barley varieties. The genomic position of each single-nucleotide polymorphism (SNP) is indicated by a blue vertical line. The coding sequence (CDS) is represented by a blue box, the untranslated region (UTR) by an orange box, and introns are denoted by green boxes.

**Figure 4:**
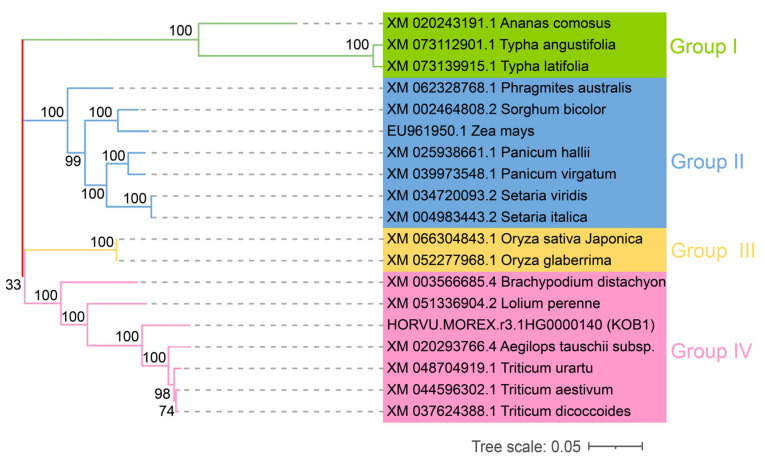
Phylogenetic analysis of candidate gene *KOB1* and its homologous sequences. The evolutionary tree was constructed using the Neighbor-Joining (NJ) method. Bootstrap values from 1000 replicates are shown at the nodes. Different colors represent distinct phylogenetic clades.

The gene *KOB1* is located approximately 89 kilobases upstream of the most significant SNP (1_1033963) and resides within a high linkage disequilibrium block (Figure 3A). Next, we analyzed the haplotypes of *KOB1* in the barley population. We identified six haplotypes of *KOB1*, with haplotype B (HapB) as the major haplotype (Figure 3C). The mean β-glucan content of haplotype E (HapE) was higher than the other haplotypes. A significantly higher β-glucan content was observed in HapE relative to the other haplotypes (* *p* < 0.05). Therefore, HapE is a favorable allele for β-content, and the introduction of the HapE allele of *KOB1* into other cultivars may help to increase β-content (Figure 3B). The gene structure of barley *KOB1* differs from that of its *Arabidopsis* ortholog, exhibiting variations in the length of coding regions and the positioning of exons. These structural divergences likely result from their distinct evolutionary paths and may underlie potential functional differentiation between the two orthologs (Figure 3C). Determining the relationships between *KOB1* gene sequences in species can deepen our understanding of the evolutionary history of individual genes. The coding sequence of barley *KOB1* was used as a query in a BLAST 2.17.0 search against the NCBI database, which revealed high sequence homology with putative glycosyltransferases from *Aegilops tauschii*, *Triticum urartu*, *Triticum aestivum*, and *Triticum dicoccoides* (Figure 4). These results suggest that *KOB1* is evolutionarily conserved within the *Triticeae*, underscoring its potential functional importance in β-glucan biosynthesis.

### 2.4. Identification of the Gene Controlling β-Glucan Content

**Figure 5:**
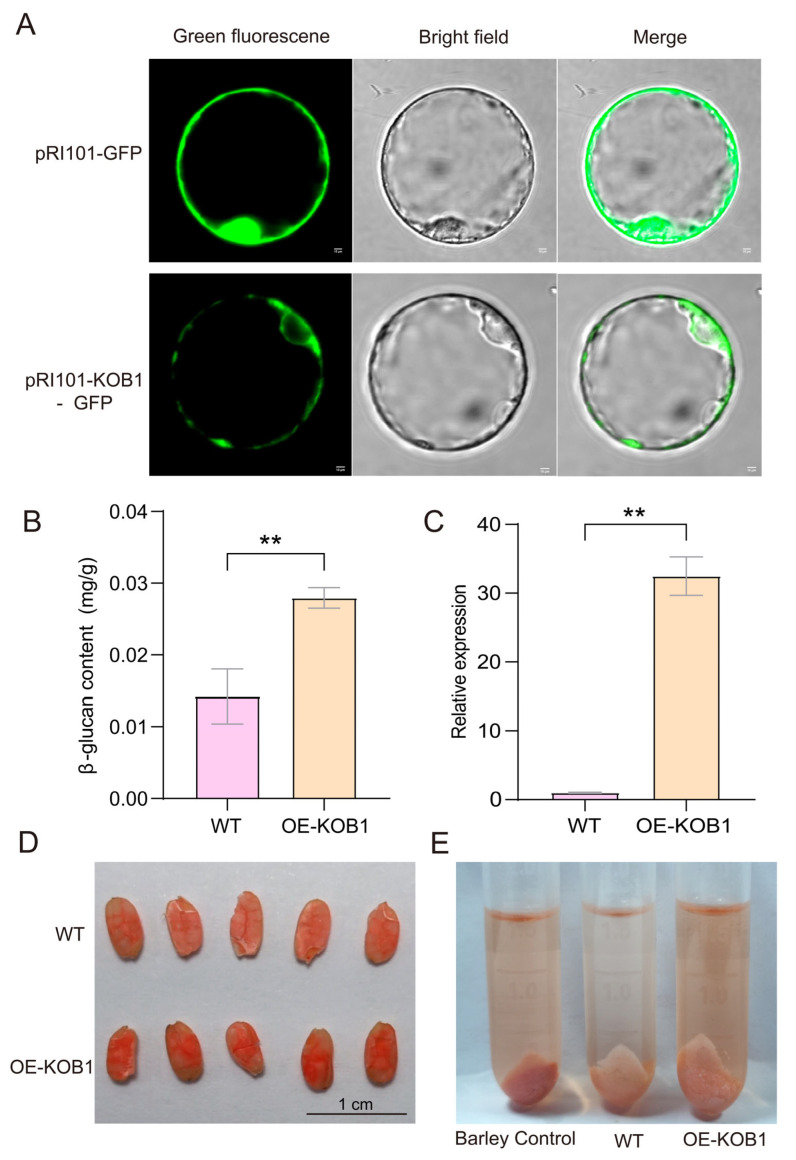
Transgenic verification of the *KOB1* gene. (**A**) Subcellular localization of *KOB1* protein in rice protoplasts. The pRI101-GFP empty vector (top) shows the typical diffuse distribution of free GFP in the cytosol and nucleus, serving as a negative control; the pRI101-KOB1-GFP fusion protein (bottom) exhibits a specific subcellular localization pattern. Green indicates GFP fluorescence. Scale bar, 10 μm. (**B**) β-glucan content in wild-type and transgenic rice grains. Asterisks indicate statistical significance by Student’s *t*-test (** *p* < 0.01). Values are the mean of three biological replicates ± SD. (**C**) Quantitative analysis of *KOB1* expression levels in wild-type and transgenic rice plants. Values are the mean of three replicates ± SD. Asterisks indicate statistical significance by Student’s *t*-test (** *p* < 0.01). (**D**) β-glucan content analysis of longitudinal grain sections via Congo Red staining. The OE-KOB1 line shows more intense red coloration in the endosperm compared to WT. Scale bar, 1 cm. (**E**) Semi-quantitative assessment of β-glucan content by Congo red staining of ground grain powders. Barley Control: the standard powder (4.1% β-glucan) supplied with a commercial β-glucan assay kit.

To validate the role of *KOB1* gene in β-glucan biosynthesis in grains, we generated an overexpression construct and produced transgenic plants in rice cultivar Zhonghua 11 (ZH11). Multiple independent transgenic lines were obtained and verified by PCR (Figures S1 and S3). Furthermore, to investigate its subcellular localization, a *KOB1*-GFP fusion construct was transiently expressed in rice protoplasts. Analysis of the subcellular localization results indicated a weaker fluorescence signal intensity compared to the GFP control. Localization experiments suggested that the signal may originate from the endoplasmic reticulum (Figure 5A and Figure S2). This observation aligns with Wolf PSORT (https://www.genscript.com/wolf-psort.html, accessed on 23 July 2025) predictions, which indicated the highest probabilities for nuclear (15.8%), mitochondrial (14.9%), and endoplasmic reticulum (14.7%) localization. Transgene-positive lines were cultivated individually, and seeds from each plant were harvested separately at maturity (Figure S4). We quantified the β-glucan content in grains from wild-type and transgenic strains with a commercial kit (GLC-1-Y, Suzhou Kemin Biotechnology, Suzhou, China). The results showed that the β-glucan content of the transgenic strain OE-KOB1 was significantly higher compared with the wild strain ZH11, which was 1.97-fold increase (*p* < 0.01, Student’s *t*-tests) than that of the wild-type rice, indicating that the gene plays an important role in increasing the β-glucan content (Table S5, Figure 5B). We observed significantly deeper Congo red staining in the endosperm of OE-KOB1 grains, showing a distinct gradient compared to the wild type (Figure 5D). Consistent with this, powder samples from the OE-KOB1 line also displayed a substantially higher staining intensity (Figure 5E). The stronger overall staining intensity in the OE-KOB1 sample corroborates the quantitative data from (B). qRT-PCR analysis demonstrated that *KOB1* expression in developing grains (5 DPA) of the transgenic line was significantly elevated, reaching a level 32.48-fold higher than that in the wild-type (Figure 5C, Table S6). We assessed tiller number, plant height, and grain dimensions in mature plants and found no significant differences between overexpression lines and the wild-type (Figure S4).

## 3. Discussions

β-Glucan is a polysaccharide macromolecule composed of glucose molecules linked by β-(1,3) glycosidic bonds and β-(1,4) glycosidic bonds, which is widely used in the fields of pharmaceuticals, nutraceuticals, and foods because of its various biological activities and pharmacological effects [30]. Garcia-Gimenez et al. reported that the β-glucan content of both wheat and rice was much lower than the β-glucan content of barley [26]. With the improvement of molecular breeding theory and technology, GWAS analysis based on linkage disequilibrium has become an important tool for crops to uncover genetic loci and mechanisms of trait variation [31,32,33]. In order to search for genes related to barley β-glucan, SNP genotyping was performed on 238 diverse barley accessions by barley 40K SNP chips, and a total of 43,930 high-quality SNP markers were obtained by quality control of polymorphic markers and GWAS analysis of multi-year experiments, and 19 significant SNP loci were screened under the condition of setting the significance threshold to −log_10(P)_ ≥ 4.64, which was associated with the significant β-glucan-associated loci. Houston et al. used the 9K SNP chips to genotype 8938 SNP markers by genos scanning DNA from 603 barley materials and detected 14 significant SNPs [19]. Some researchers obtained 191,098 SNP markers by SNP genotyping on 87 barley materials through the illumina Hiseq 2500 platform, and seven SNPs significantly associated with β-glucan content were obtained by GWAS analysis [20]. Our study identified a greater number of significant SNP loci compared to previous reports, which can likely be attributed to the use of a larger natural population and a higher density of SNP markers enabled by high-throughput genotyping. These findings demonstrate that expanding both population size and marker density enhances the detection power and accuracy of genome-wide association studies. Several studies have reported the identification of two glycosyltransferases through GWAS of β-glucan content in wild barley accessions [34]. Geng et al. conducted a GWAS on an international barley core collection and identified several significant loci, particularly on chromosomes 1H and 4H [27]. In our study, we also identified a novel and highly significant locus on chromosome 1H. Furthermore, while Geng et al. proposed several candidate genes, including glycosyltransferases, our work builds upon this by not only identifying a key glycosyltransferase-encoding gene (KOB1) but also providing functional validation through transgenic overexpression—thereby strongly confirming its pivotal role in β-glucan biosynthesis. Zhang et al. employed quantitative proteomic analysis to investigate proteins associated with β-glucan accumulation in barley grains, revealing that the upregulation of amylase, β-glucosidase, and glycosyltransferase correlated with β-glucan synthesis. This finding aligns with our results regarding the involvement of glycosyltransferase in β-glucan biosynthesis [35].

Garcia-Gimenez et al. quantified the transcript abundance of genes *HvCslF6*, *HvCslF9*, and *HvGlb1* across six grain developmental stages (3–5, 8–10, 15, 24–26, 32, and 38 days post-anthesis) in four barley cultivars. They observed that the expression patterns of these genes varied significantly among cultivars at different developmental stages [26]. Through transcriptome analyses of two barley genotypes differing substantially in seed β-glucan content, Geng et al. demonstrated distinct temporal accumulation patterns of β-glucan during seed development (7, 14, 21, and 28 DPA) [28]. Their analysis further revealed that genes encoding UDP-glycosyltransferase superfamily proteins were up-regulated in both genotypes, indicating a positive role in regulating β-glucan accumulation [36]. In our study, analysis of the expression pattern of the glycosyltransferase-encoding gene *KOB1* during seed development revealed elevated expression at the 5 DPA, LOD, and RAC stages, which is similar to the pattern observed in previous studies.

Glycosyltransferases are believed to play an important role in β-glucan synthesis [36,37]. In Arabidopsis, the *KOB1* glycosyltransferase functions in cellulose biosynthesis and plasmodesmal permeability [29]. In contrast, its barley homolog *KOB1* regulates β-glucan content, revealing a distinct role that likely represents an evolutionary adaptation in grasses. Our localization studies reveal that the barley KOB1 protein and its Arabidopsis ortholog exhibit diverged localizations within the secretory pathway, indicating potential functional diversification between species. We propose a model in which *KOB1* acts as an auxiliary protein within the β-glucan synthase complex, potentially modulating its activity or substrate supply to explain the increase in β-glucan content. A critical test of this model will be to directly probe for physical interaction between *KOB1* and the core synthase in future studies. Glycosyltransferases are responsible for catalyzing the glycosyl transfer reaction during the synthesis of β-glucan, and the expression of genes encoding glycosyl hydrolases increase significantly during this process [38]. It has also been found that some other enzymes involved in the β-glucan synthesis process also significantly affect the content of β-glucan [39]. The gene *HORVU.MOREX.r3.3HG0298680* encodes the MYB transcription factor, which modulates the transcription level of CslF6 in barley grains, thereby altering the β-glucan content of barley grains [40]. The gene *HORVU.MOREX.r3.3HG0300770* encodes the ABI5 transcription factor, which plays an important role in the development of barley grains. The ABI5 transcription factor mediates upregulation of ABA-responsive genes and affects the functional changes in the cells of barley aleurone layer, which potentially influences in the content of barley grains β-glucan [41]. Song et al. noted that with the advent of haplotype region mapping, one can effectively select SNPs and haplotype regions for optimal association analysis [42]. Liu et al. discovered relevant dominant haplotypes and haplotype combinations through haplotype analysis of candidate genes related to grain length and grain weight, which provide excellent genetic resources for rice molecular breeding [43]. In this study, HApE was identified as a favorable haplotype, which may provide a molecular basis for the introduction of dominant haplotypes to improve breeding efficiency in molecular breeding of barley. Zhang et al. investigated the heterologous expression of the maize D14 gene in Arabidopsis thaliana and found that the gene provided enhanced plant drought tolerance [44]. *WRKY* genes are involved in plant development, stress response. Some researchers overexpressed the maize *WRKY114* gene in rice and found that it reduces plant height by regulating the biosynthesis of GA [45].

Geng et al. identified a gene encoding β-glucosidase 3 through GWAS and generated an *HvBGlu3* knockout mutant via CRISPR/Cas9-mediated editing, thereby confirming the role of GH family genes in the synthesis and accumulation of β-glucans in barley grains [46]. We heterologous expressed the *KOB1* gene from barley in rice to study its function and found that its overexpression significantly increased β-glucan in seeds (T_1_). This study, through an integrated strategy, establishes *KOB1* as a high-confidence candidate gene for β-glucan content. However, we emphasize that the heterologous validation in rice, while indicative of a functional capacity, marks the initial step rather than a definitive functional assignment. Future research must now focus on barley, utilizing gene editing and stable transformation to confirm its in planta function, define its precise role in β-glucan biosynthesis, and identify its interaction partners within the barley synthetic complex. Discrepancies between our findings and earlier reports on the genetic basis of β-glucan synthesis underscore the dynamic complexity of this process and open new avenues for investigating its genetic mechanisms [4,37]. By identifying robust loci and validating a key candidate gene, this study provides a foundational resource for future molecular breeding strategies aimed at the targeted modification of β-glucan levels in barley.

## 4. Materials and Methods

### 4.1. Plant Materials

A global collection of 238 barley accessions was used for GWAS. This panel included cultivars and landraces from worldwide sources, ensuring a broad genetic base for analysis (Table S1). The 238 barley germplasm materials used in this study were obtained from the College of Agronomy at Gansu Agricultural University and planted at the University’s Huangyang Town Experimental Station in Wuwei, Gansu Province, China. The transgenic rice material was the rice variety ZH11, grown at Gansu Agricultural University. Seeds were harvested when the kernels were ripe, threshed manually and stored at 4 °C in a seed cabinet.

### 4.2. Genome-Wide Association Studies and Candidate Gene Identification

Genotyping of 238 barley natural accessions with the Barley 40K SNPs array [47] yielded 171,001 SNP markers. Quality control was then performed using Plink1.9 [48] software, resulting in 114,652 high-quality SNPs. Guided by the QQ plot, we selected the model demonstrating optimal false-positive rate control as the final association analysis outcome. Using LD decay patterns, we defined candidate genomic regions as 300 kb intervals flanking significant loci to identify candidate genes [49]. Thirty-seven candidate genes identified by GWAS analysis of β-glucan content and optimal best linear unbiased prediction values [50] of barley populations. These candidate genes were functionally annotated using the barley genome database (https://plants.ensembl.org/Hordeum_vulgare/Info/Index, accessed on 4 March 2024), the Arabidopsis information resource (https://www.arabidopsis.org/, accessed on 4 March 2024) and the NCBI database (https://www.ncbi.nlm.nih.gov/, accessed on 4 March 2024). We prioritized genes based on annotation and predicted function, focusing on those encoding enzymes directly involved in polysaccharide biosynthesis or those with homology to known (1,3;1,4)-β-glucan synthases (*CSLF* and *CSLH* genes) from other species. The barley expression database website (http://barleyexp.com/, accessed on 6 March 2024) was used to analyze the expression of barley at different developmental stages (SRA BioProject: PRJEB14349).

### 4.3. Haplotype-Based Association Analysis

Haplotype analysis of selected candidate genes was performed using the software Haploview 4.2 [51] in combination with phenotypic data to identify favorable haplotypes. The presence of interlocking blocks within the selected genes was analyzed and combined with phenotypic data to further determine the linkage. Linkage disequilibrium analysis of candidate genes was performed and visualized using the software LDBlockShow 1.35 [52]. Finally, the candidate genes were analyzed for homology by NCBI blast comparison to obtain homologous gene sequences. Gene sequences were sequence aligned by MEGA11 [53] and the homology tree was beautified by the iQTL website (https://itol.embl.de/, accessed on 10 May 2025).

### 4.4. Functional Characterization of Candidate Gene

To investigate the biological function of the candidate gene *KOB1*, molecular cloning and heterologous expression were carried out using the rice cultivar Zhonghua 11 [54]. The reference coding sequence (CDS) of *KOB1* was obtained from Ensembl Plants (https://plants.ensembl.org/Hordeum_vulgare/Info/Index, accessed on 11 March 2024), and gene-specific primers were designed accordingly (Tables S3 and S4). Total RNA was extracted from barley seedlings using TRIzol reagent. PCR amplification was conducted with Phanta Max Super-Fidelity DNA Polymerase (Vazyme Biotech Co., Ltd., Nanjing, China) under the following conditions: Initial denaturation at 95 °C for 30 s; 39 cycles of 95 °C for 15 s, 55 °C for 15 s, 72 °C for 1 min; final extension at 72 °C for 5 min. Amplification of the barley *KOB1* gene’s full-length CDS was performed via PCR.

### 4.5. Subcellular Localization Vector

To determine the subcellular localization of the candidate gene *KOB1*, the coding region lacking the stop codon was amplified by PCR. The resulting product was cloned into the SalI site of the pRI101-GFP vector to generate a GFP fusion construct under the control of the 35S promoter. The integrity of the fusion construct was confirmed by sequencing using the primers 35S-F (5′-GACGCACAATCCCACTATCC-3′) and GFP-JR (5′-GGGTGAGCTTGCCGTAGGTG-3′). The verified plasmid was then introduced into isolated rice protoplasts (devoid of chloroplast autofluorescence) via polyethylene glycol (PEG)-mediated transfection [55]. After incubation at 28 °C for 48 h in darkness, fluorescence signals were captured using a Nikon C2-ER laser scanning confocal microscope [56].

### 4.6. Overexpression Vector

The full-length coding sequence of *KOB1* was cloned into the BamHI site of the plant expression vector pCambia2301-JC, placing it under the control of the CaMV 35S promoter. This vector, a derivative of pCambia2301, carries both a kanamycin selectable marker for bacterial selection and a hygromycin B selectable marker for plant selection. The sequence integrity of the overexpression construct was verified by DNA sequencing, employing the following primers: 35S-F, 5′-GACGCACAATCCCACTATCC-3′; and M13-48, 5′-GAGCGGATAACAATTTCACAC-3′. The recombinant plasmid was introduced into Agrobacterium tumefaciens strain GV3101 via freeze–thaw transformation. For stable rice transformation, embryogenic calli of ZH11 were co-cultivated with the transformed Agrobacterium [57]. Following co-cultivation, regenerated plants were selected on medium containing 50 mg/L hygromycin B [58]. Multiple independent transgenic lines were obtained for gene function validation. The primers used for gene cloning and vector construction are provided in Supplementary Table S4.

### 4.7. Transgenic Plant Verification and Phenotype Measurement

Genomic DNA extracted from transgenic leaves was analyzed by PCR using vector-specific primers to confirm the presence of the transgene. Transgenic lines showing clear 400 bp vector-band amplification were advanced for phenotyping. Both the transgenic lines and the wild-type ZH11 controls were cultivated as individual plants in a controlled environment under identical conditions. Upon reaching physiological maturity, grains were manually harvested on a per-plant basis and stored separately in dedicated seed cabinets. To semi-quantitatively assess β-glucan, longitudinally sectioned mature barley grains were stained with 0.1% (*w*/*v*) Congo red [59,60,61]. In parallel, staining of grain powder provided a complementary assessment of overall content. Seed β-glucan content was quantified using a commercial assay kit (GLC-1-Y, Suzhou Kemin Biotechnology) on powder from thirty ground and sieved (40-mesh) seeds per plant line (T_1_), with three analytical replicates. The relative expression of *KOB1* in developing grains (5 DPA) was quantified by qRT-PCR in transgenic lines compared to the wild-type, using the *Actin* gene for normalization across three replicates per line. Primer sequences were F: 5′-TCCTCCGTGGAGAAGAGCTA-3′; R: 5′-GCAATGCCAGGGAACATAGT-3′.

## Data Availability

The data associated with this study are included in this article and its Supplementary Information Files. All other data are available from the corresponding author upon reasonable request.

## Supplementary Materials

The following supporting information can be downloaded at: https://www.mdpi.com/article/10.3390/plants14213269/s1. Figure S1: Construction of barely *KOB1* gene expression vector and PCR verification. (A) Schematic diagram of expression vector construction. (B) PCR verification of transformed bacterial colonies. Lanes 1–8 represent individual colonies. The expected size of the PCR product was approximately 1.6 kb. Clone 3 (lane 3), confirmed by sequencing, was selected for all downstream experiments. M, 2 kb marker; Figure S2: Construction and verification of the barley *KOB1* localization vector. (A) Schematic diagram of the vector construct. (B) PCR verification of transformed colonies. Lanes 1–6 represent individual colonies. Clone 3 (lane 3), which was confirmed by sequencing, was selected for subsequent experiments. M, 2 kb marker; Figure S3: PCR analysis confirms the generation of *KOB1* transgenic rice lines. Amplification of a ~400 bp fragment corresponding to the hygromycin selection gene is shown. WT, wild type; +, A. tumefaciens positive control; −, H_2_O negative control; lanes 5–14, positive transgenic lines; Figure S4: Establishment and preliminary phenotyping of OE-KOB1 transgenic rice. (A–E) Tissue culture and regeneration stages. (F) A regenerated T0 plant. (G) Comparison of T1 seeds from wild-type and hemizygous T0 OE-KOB1 lines; Table S1: Description of the 238 barley germplasm materials used in this study; Table S2: Expression level of candiate gene; Table S3: *(HORVU.MOREX.r3.1HG0000140) KOB1* gene coding sequence; Table S4: The primers used for genetic transformation and transgene validation experiments; Table S5: β-glucan content in rice grains; Table S6: KOB1 Expression Level (qRT-PCR).
