# Supplementary material for: The Barley Glycosyltransferase Gene KOB1 Implicated in β-Glucan Biosynthesis by a Genome-Wide Association Study
Source: Plants (Basel). 2025 Oct 26;14(21):3269. doi: 10.3390/plants14213269 (PMC12610209; doi:10.3390/plants14213269)
Supplement: Supplementary file 1 [file plants-14-03269-s001.zip › Supplementary Fig.pdf]

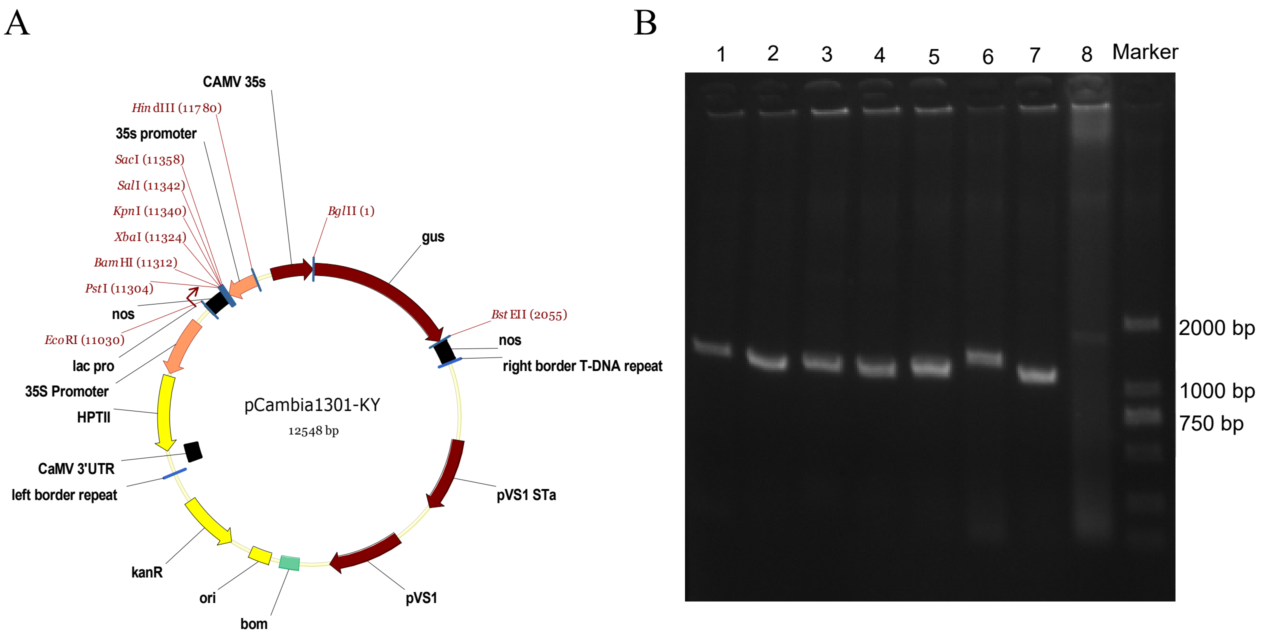

Figure S1. Construction of barely *KOB1* gene expression vector and PCR verification. (A) Schematic diagram of expression vector construction. (B) PCR verification of transformed bacterial colonies. Lanes 1-8 represent individual colonies. The expected size of the PCR product was approximately 1.6 kb. Clone 3 (lane 3), confirmed by sequencing, was selected for all downstream experiments. M, 2 kb marker.

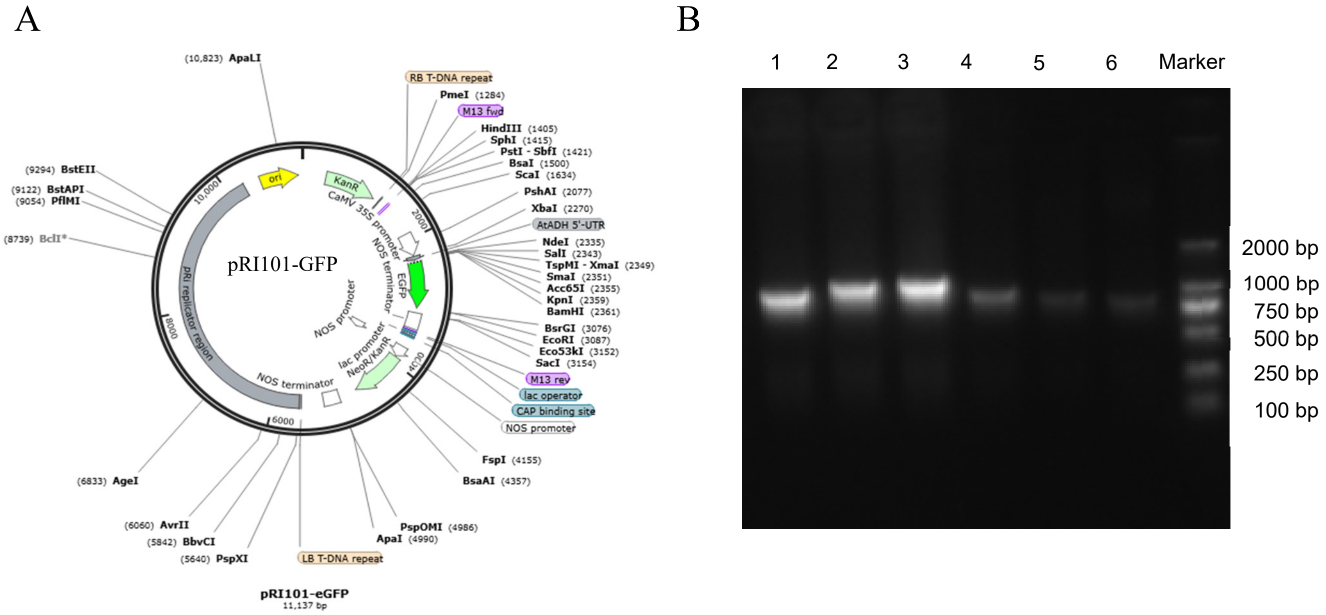

Figure S2. Construction and verification of the barley *KOB1* localization vector. (A) Schematic diagram of the vector construct. (B) PCR verification of transformed colonies. Lanes 1-6 represent individual colonies. Clone 3 (lane 3), which was confirmed by sequencing, was selected for subsequent experiments. M, 2 kb marker.

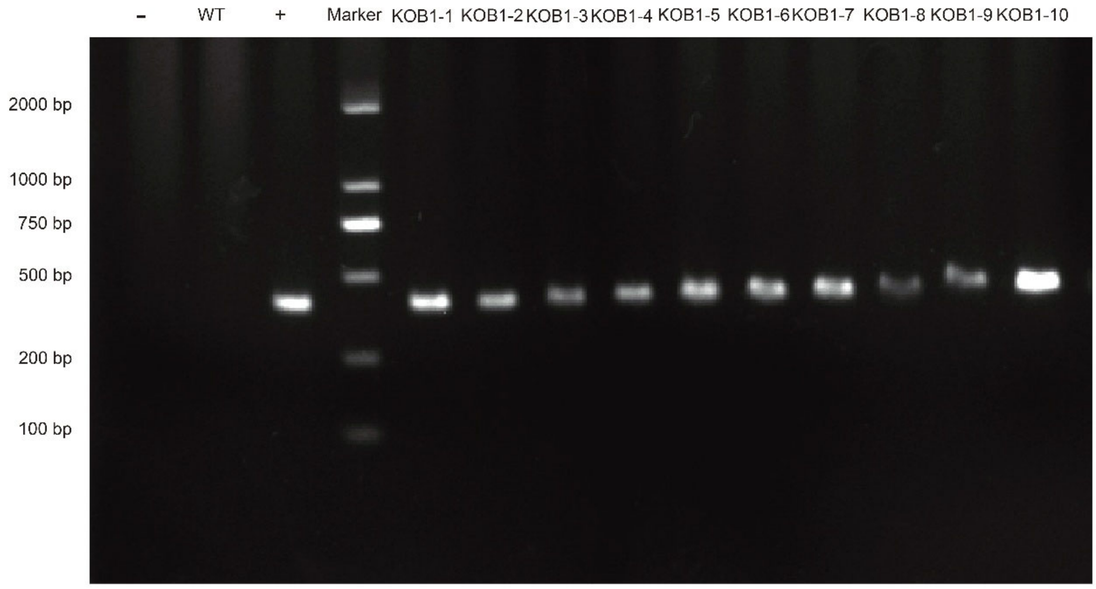

Figure S3. PCR analysis confirms the generation of *KOB1* transgenic rice lines. Amplification of a ~400 bp fragment corresponding to the hygromycin selection gene is shown. WT, wild type; +, A. tumefaciens positive control; –, H_2O negative control; lanes 5–14, positive transgenic lines.

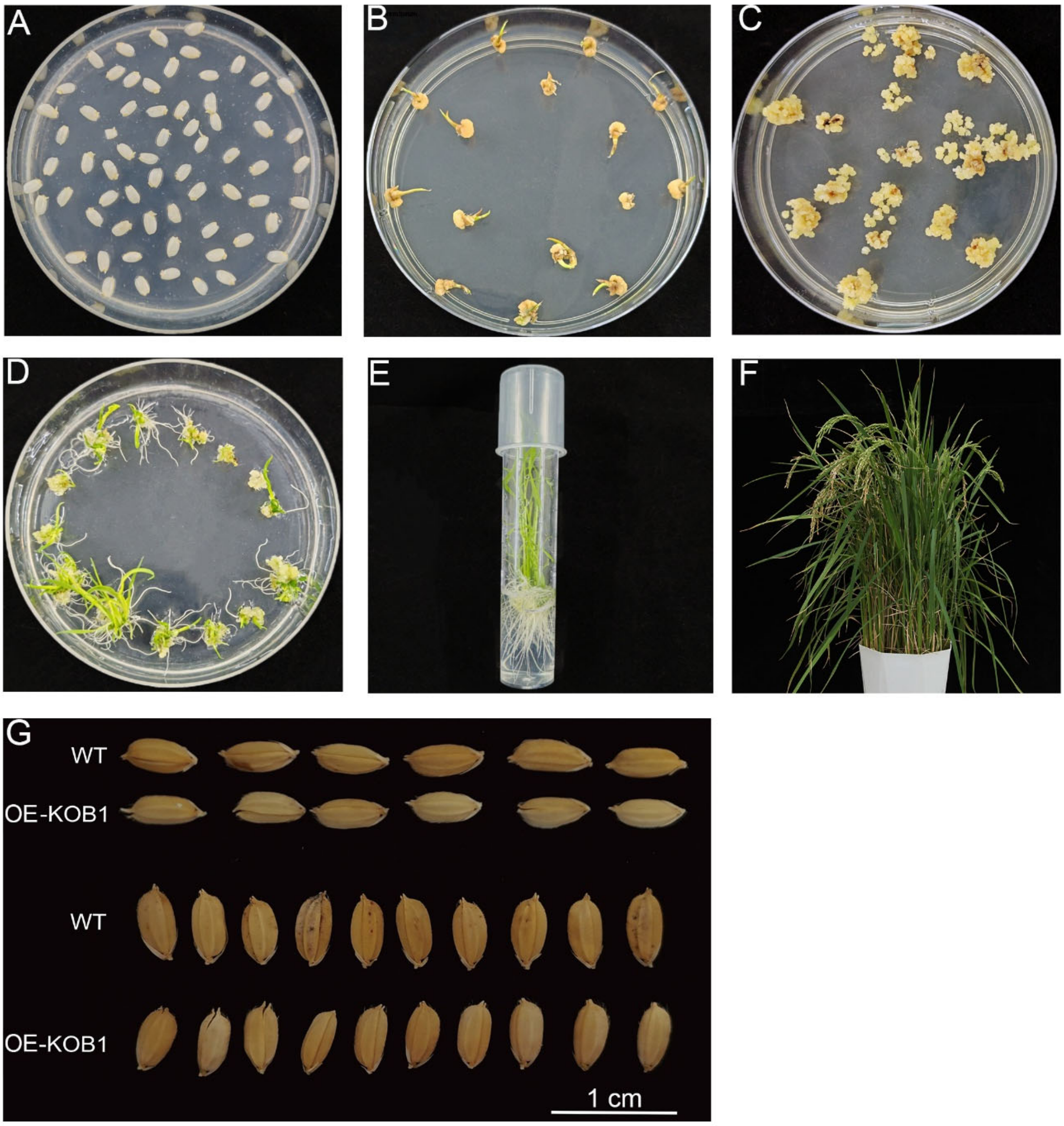

Figure S4. Establishment and preliminary phenotyping of OE-KOB1 transgenic rice. (A-E) Tissue culture and regeneration stages. (F) A regenerated T0 plant. (G) Comparison of T1 seeds from wild-type and hemizygous T0 OE-KOB1 lines.
